# Mapping of the country-wide prevalence of non-malarial febrile illnesses in areas with varying malaria transmission intensities in Mainland Tanzania

**DOI:** 10.12688/gatesopenres.16391.1

**Published:** 2026-07-03

**Authors:** Salehe S. Mandai, Angelina J. Kisambale, Daniel A. Petro, Catherine Bakari, Gervas A. Chacha, Rule Budodo, Rashid A. Madebe, Dativa Pereus, Daniel P. Challe, Ramadhani Moshi, Ruth A. Mbwambo, Grace K. Kanyankole, Sijenunu Aaron, Daniel Mbwambo, Stella Kajange, Samwel Lazaro, Ntuli Kapologwe, Celine I. Mandara, Misago D. Seth, Deus S. Ishengoma

**Affiliations:** 1 National Institute for Medical Research, Dar es Salaam, Tanzania; 2 University of Dar es Salaam, Dar es Salaam, Tanzania; 3 National Institute for Medical Research, Tanga Research Centre, Tanga, Tanzania; 4 National Malaria Control Program, Dodoma, Tanzania; 5 President’s Office-Regional Administrative Local Government, Dodoma, Tanzania; 6 Directorate of Preventive Services, Ministry of Health, Dodoma, Tanzania

**Keywords:** Malaria, Fever, non-malaria febrile illness, Plasmodium falciparum, Tanzania

## Abstract

**Background:**

Despite the declining malaria burden in endemic countries, reports of febrile episodes do not align with the current patterns of malaria transmission. This highlights the presence of non-malarial febrile illnesses (NMFIs), which are difficult to distinguish from malaria. This study assessed the country-wide prevalence of NMFIs and their patterns across various malaria transmission settings in Mainland Tanzania.

**Methods:**

A cross-sectional study recruited patients aged ≥ 6 months from 86 health facilities in all 26 regions of Tanzania. All patients were tested for malaria using rapid diagnostic tests (RDTs). For this study, patients with fever who tested negative by RDT were operationally classified as having NMFIs and their prevalence was determined accordingly, Logistic regression was used to determine factors associated with NMFIs.

**Results:**

Of the 18,568 patients tested, 8,273 (44.6%) were considered to have NMFIs because they tested negative by RDT. Higher prevalence of NMFIs occurred in females (45.8%) than in males (42.8%), adults (aged ≥ 15 years, with 50.6%) compared to under-fives (42.6%) and school children (aged 5 -< 15 years, 34.3%), and in very low (65.5%) compared to high transmission areas (34.5%). NMFIs were significantly more likely in females than in males (aOR = 1.18, 95% CI = 1.10–1.26), under-fives (aOR = 1.60, 95% CI = 1.47–1.76) and less likely in high transmission areas (aOR = 0.11, 95% CI = 0.08–0.13), and school children (aOR = 0.70, 95% CI = 0.64–0.77).

**Conclusion:**

The findings show a high prevalence of NMFIs overall, and higher prevalence and odds of NMFIs in females and individuals from low and very low transmission areas. These groups should be targeted with appropriate point-of-care tests and treatment strategies.

## Introduction

Although febrile illnesses are the most common reasons for seeking medical care globally, ^1^^,^^2^ diagnosis and management of these illnesses are challenging due to their diverse causes and the general nature of their symptoms, which are hard to distinguish clinically. ^3^^,^^4^ In malaria-endemic settings, most febrile cases are often considered to be malaria, due to the similarity of their clinical presentation, leading to under-reporting and mismanagement of other causes of fevers. ^5^^,^^6^ However, recent advancements in diagnostic technologies, along with intensified control efforts, have shed new light on the broader landscape of febrile illnesses, revealing a complex interplay of pathogens contributing to fevers in endemic areas. ^7^ The widespread use of malaria rapid diagnostic tests (RDTs) has been pivotal in this shift. These tests swiftly and rapidly confirm malaria infections by detecting parasite proteins, aiding healthcare providers in making confirmatory diagnoses and distinguishing malaria from non-malarial fevers. ^8^ In practice, there are limited point-of-care (POC) tests for other pathogens that cause non-malaria fevers ^9^ and thus, the causes of other pathogens responsible for fevers apart from malaria are not routinely detected. This leads to poor management of cases without malaria, as well as a missed opportunity to capture other fever-causing pathogens. ^10^ With the recent decline of the malaria burden in areas that were hyper-endemic for many years, failure to detect the different causes of fevers in patients with negative RDT results is an emerging dilemma for service providers. Thus, urgent actions are required to develop POC tests for multiple pathogens and algorithms for their effective management.

In recent years, Tanzania, like many other endemic countries, has witnessed a remarkable decline in malaria transmission and disease burden in different areas. ^11^ However, the decline in malaria cases does not correspond to the decrease in febrile patients in areas of different malaria endemicity. In some locations, the proportion of patients with fever but without malaria parasites has remained unchanged or increased significantly. ^12^ This discrepancy highlights the increasing recognition that many febrile illnesses previously treated presumptively as malaria are actually of non-malarial origin. This is especially noticeable in areas with low malaria transmission, where a large proportion of individuals with fever test negative for malaria upon visiting health facilities. Conversely, in high transmission settings, a positive RDT result does not always indicate that malaria is the only cause of fever, ^13^^,^^14^ because individuals may have developed naturally acquired immunity to malaria, ^15^^,^^16^ and the presenting symptoms may result from co-infections with different pathogens. Therefore, a comprehensive, evidence-based strategy with a clear management algorithm is urgently needed for fevers not caused by or occurring concurrently with malaria infections.

While it is challenging to determine whether there is an increase in NMFIs, more work should be done to enhance our understanding of the epidemiology of these conditions and pathogens causing NMFIs. Recent reviews revealed that NMFIs are widespread in Africa, and most of them are dominated by bacterial and viral aetiologies. ^17^^–^^19^ A recent study in Nigeria found that 37.0% of febrile cases were not caused by malaria. Among these cases, 54.7% were due to viral infections, while bacterial infections caused 32.1% of the infections. ^20^ Similarly, a study in Senegal identified other causes of fever in 23.0% of febrile cases. ^7^ In another study conducted in Guinea-Bissau, respiratory viruses were the most common pathogens, detected in 58.9% of non-malarial febrile cases, followed by bacteria, which were found in 22.6% of the cases. ^21^

In contrast to malaria, other causes of febrile illnesses are frequently unnoticed in many regions of Sub-Saharan Africa due to the lack of comprehensive guidelines for managing febrile cases when malaria is not the cause. ^6^^,^^22^ This neglect stems from limited human and financial resources, ^23^ and a lack of understanding and insufficient appreciation of the public health burden posed by NMFIs. ^24^ The limited awareness is further compounded by inadequate training and diagnostic capacity, which hinders the ability of healthcare workers to correctly identify and appropriately manage NMFIs. ^25^^–^^27^ In many regions, diagnostic tools for these illnesses are either unavailable or underutilised due to logistical challenges, leading to presumptive treatment. As a result, authorities are compelled to prioritise diseases deemed more threatening to public health, while NMFIs are overlooked due to insufficient evidence of their true burden. ^28^ Subsequently, the lack of evidence-based information on the burden of these illnesses further complicates the situation, leading to disregard for the potential threat of NMFIs. ^29^ This cycle of neglect continues as limited data on NMFIs hampers the development of effective policies and guidelines, and diminishing incentives to invest in diagnostic capacity. In addition, the decline of malaria is found to be associated with an increase in antibiotic prescriptions, ^30^^,^^31^ highlighting the potential presumptive treatment of NMFI cases with antibiotics, which is inappropriate and could result in antibiotic resistance, a growing global concern. ^32^^,^^33^

Healthcare workers in Tanzania, as elsewhere, face challenges in managing febrile patients with negative malaria RDTs. This highlights the urgent need to better understand the epidemiology and burden of non-malarial febrile illnesses. Although previous studies conducted in Tanzania focused on identifying aetiological agents of NMFIs at selected sites, the extent of the burden and distribution patterns of NMFIs remain poorly understood. This study utilised the largest data set generated by the ongoing project on molecular surveillance of malaria in Mainland Tanzania (MSMT), ^34^^–^^36^ and aimed to map the country-wide prevalence and distribution patterns of NMFIs across various regions located in different malaria transmission strata. The findings from this study will inform future research and healthcare planning, and potentially improve the diagnosis and management of febrile illnesses beyond malaria, in areas of varying endemicity in the context of the ongoing epidemiological transition of malaria transmission intensities.

## Materials and methods

### Study design

This was a health facility-based cross-sectional study that was conducted in all 26 regions of Mainland Tanzania. The survey was conducted under the MSMT project, which focuses on mapping parasite populations, and drug and diagnostic resistance (by detecting the status of histidine-rich protein 2/3 *(hrp2/3)* gene deletions) as described elsewhere. ^34^^–^^38^

### Selection of study sites in all 26 regions of Mainland Tanzania

**Figure 1.**
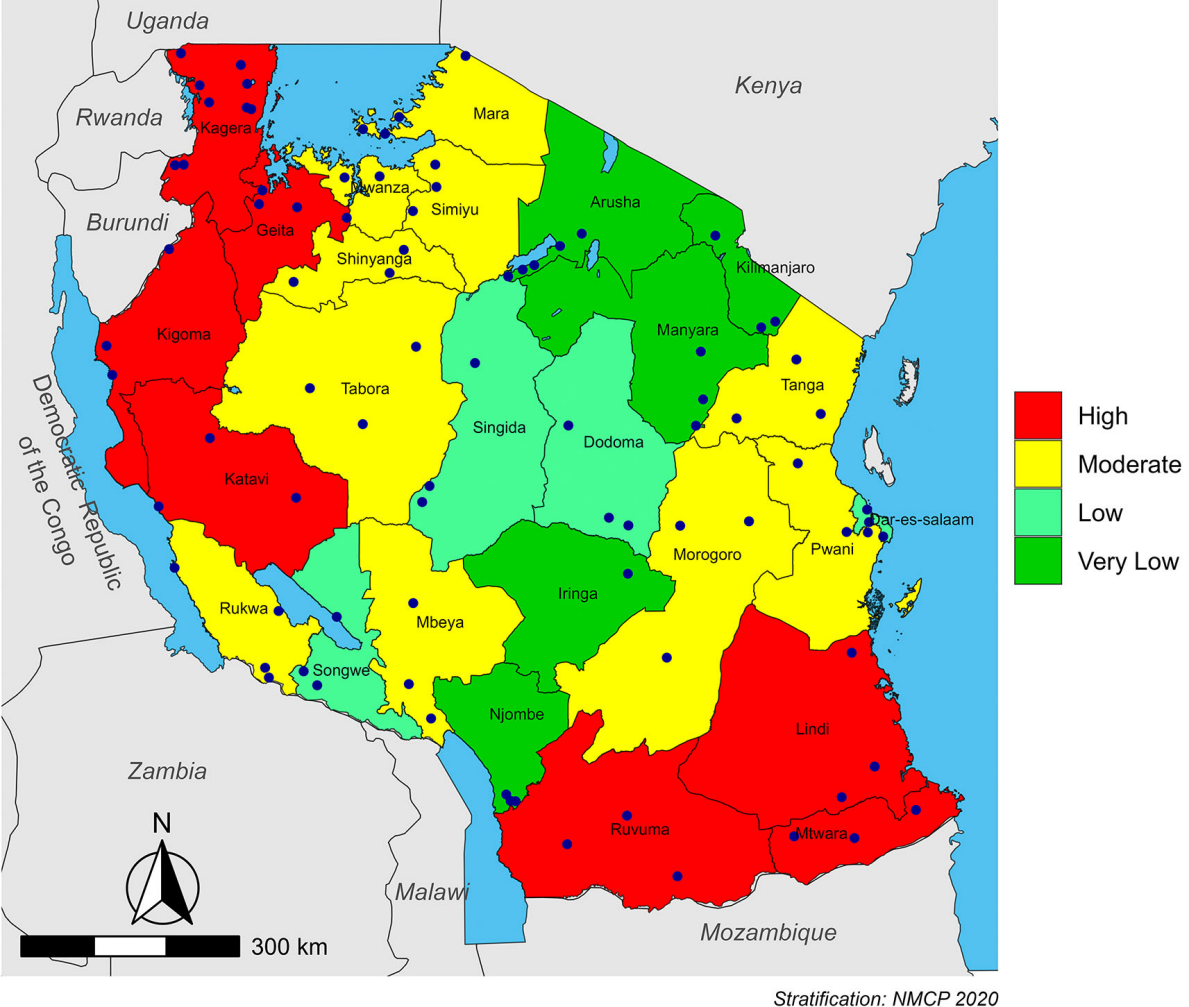
Map showing the 26 regions of Mainland Tanzania, malaria transmission strata, and the health facilities (blue dots) where the data were collected.

This was the first-ever survey of the MSMT to cover all 26 regions of Mainland Tanzania, and it was conducted at primary HFs compared to national community surveys, such as school parasitological surveys, ^39^ malaria indicator surveys ^40^^–^^42^ and demographic and health surveys. ^11^ The regions which were covered included Arusha, Dar es Salaam, Dodoma, Geita, Iringa, Kagera, Katavi, Kigoma, Kilimanjaro, Lindi, Manyara, Mara, Mbeya, Morogoro, Mtwara, Mwanza, Njombe, Pwani, Rukwa, Ruvuma, Shinyanga, Simiyu, Singida, Songwe, Tabora, and Tanga ( Figure 1). Based on the 2022 malaria stratification by the National Malaria Control Programme, the regions were categorised into four strata: high (10 regions), moderate (8 regions), low (5 regions), and very low malaria transmission intensities (3 regions) (NMCP, unpublished data). In the high transmission stratum, the regions included Geita, Kagera, Katavi, Kigoma, Lindi, Mtwara, and Ruvuma. The regions in the moderate stratum were Mara, Mbeya, Morogoro, Mwanza, Pwani, Rukwa, Simiyu, Shinyanga, Tabora, and Tanga, while the low transmission stratum had Dar es Salaam, Dodoma, Singida and Songwe, and the very low transmission stratum had Arusha, Iringa, Kilimanjaro, Manyara, and Njombe regions ( Figure 1).

In all 26 regions, 86 health facilities (HFs) were selected and involved in this study. In the 10 regions (Dar es Salaam, Dodoma, Kagera, Kilimanjaro, Manyara, Mara, Mtwara, Njombe, Songwe, and Tabora) that were covered by the MSMT project in 2021, the selection was made within the 10 HFs that were sampled in 2021. Out of these 10 HFs, three facilities were purposively selected for the 2023 survey, with a preference for the facilities that were also covered in 2022. The process of selecting the HFs that were covered by the MSMT surveys in 2021 has been previously described. ^34^^,^^38^

In each of the remaining 16 regions, three HFs from preferably three administrative councils were purposively selected to represent the diverse parts of the region and to ensure the region was appropriately represented. The selection process was done with the support of the teams from the regional and district medical officers. Initially, the data of each region was assembled from the District Health Information System 2 (DHIS2) and assessed to determine councils and HFs that reported a minimum of 50 malaria patients per month between December 2022 and April 2023. These were considered to have the capacity to recruit the required number of patients (which was 100 patients) within three months, intending to complete data and sample collection before the end of the malaria transmission season in July and August 2023. After selecting the councils and HFs with a sufficient number of patients, the target councils were selected to ensure their distribution forms a triangular spread across the region. The selection of HFs in the region/council also considered the location of facilities, which were selected in the neighbouring region to avoid over-sampling in a similar geographic area, in case selection of a facility for sampling had already been done in other regions close to it. Thereafter, the HFs were selected by considering the availability of sufficient staff to ensure that participation in the project did not impact the provision of health services to their clients. The HFs were finally selected if they were reachable by road, especially during the rainy season, because the study was done before, during, and after the long rainy season, which normally occurs between March and June.

In all regions, three councils (with one facility in each council) were involved in the survey, except for Kagera (7 Councils and 10 HFs), Kilimanjaro, Manyara, and Singida region (two councils/region), and Iringa and Arusha (one council in each region). The low number of councils in Arusha, Iringa, Kilimanjaro, Manyara, and Singida was due to low transmission intensities of malaria in these regions. Therefore, very few HFs in these regions were capable of recruiting a minimum sample of 100 patients with RDT-positive results, which was an important requirement for a site to be selected by the MSMT project. The inclusion of more councils and HFs in Kagera was due to intensive surveillance, which is currently going on in the region following the detection and confirmation of artemisinin partial resistance (ART-R). ^43^^–^^45^

### Study population and participant enrolment

The study recruited outpatients aged 6 months and above who presented at the HFs with a febrile illness suspected to be uncomplicated malaria, based on a history of fever and/or other symptoms of malaria within the previous 48 hours, and who resided in the catchment area of the selected HFs. Before recruitment, trained health facility staff who were involved in the survey provided information about the study to all outpatients and asked eligible patients for their consent to participate. All patients who met the inclusion criteria were invited to meet the study staff, who obtained informed consent and finalised the enrolment process. Consenting was done as previously described ^38^ and patients who did not consent to participate or were not residents of the study area were excluded. In addition, patients with life-threatening illnesses such as severe malaria and other conditions that required immediate care, including impaired consciousness, convulsions, and respiratory distress, were excluded from the study.

### Data collection

**Figure 2.**
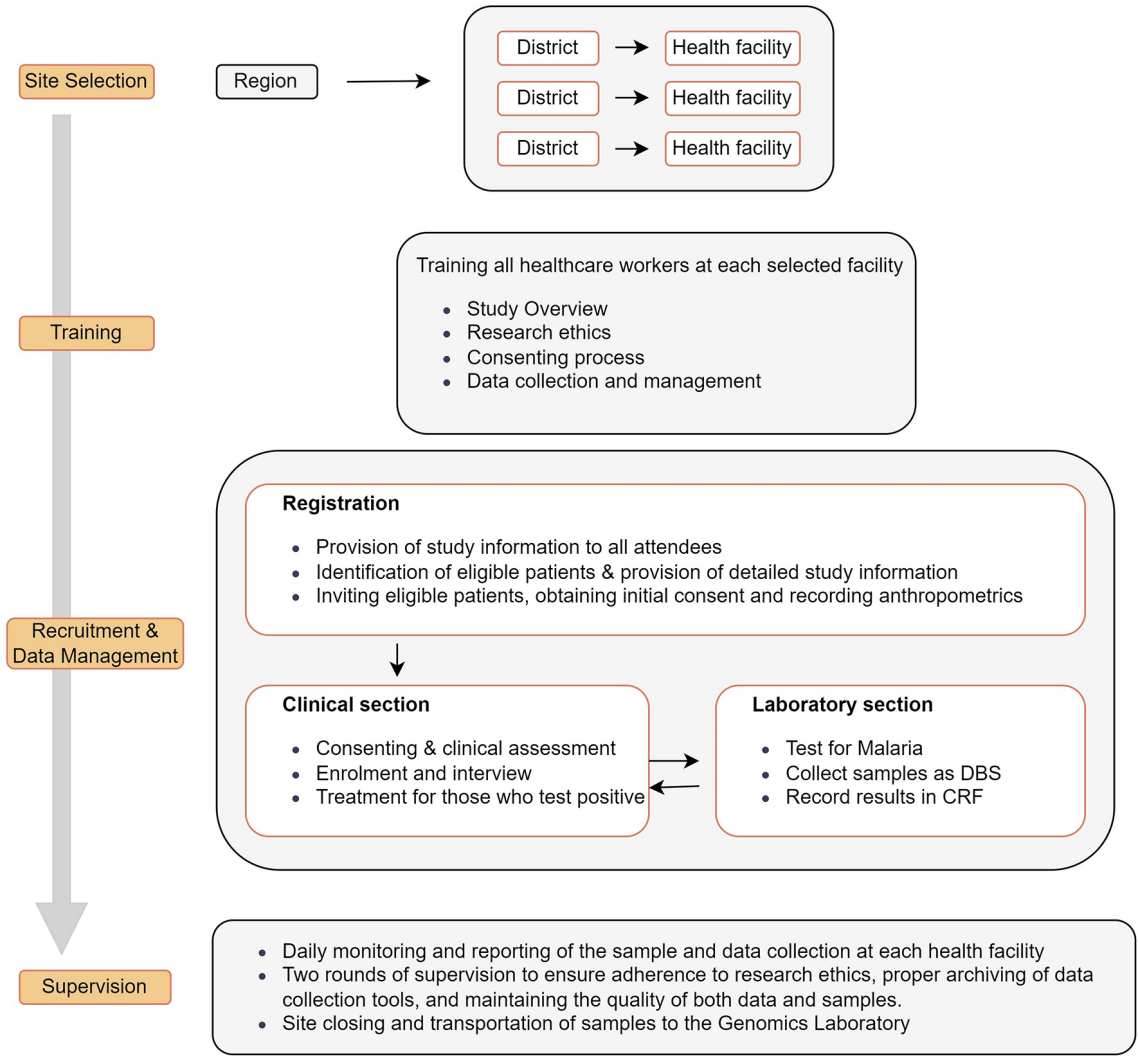
Schematic presentation of the selection of districts, health facilities, and data collection process.

For this study, all febrile patients who were recruited at the HFs while seeking care and tested RDT-negative for malaria were considered to have NMFIs. For all eligible patients, demographic, anthropometric, and parasitological data were collected after providing a consent, as shown in Figure 2. The data were collected by trained staff who participated in the 2021 or 2022 surveys of the MSMT project in the 10 regions of Dar es Salaam, Dodoma, Kagera, Kilimanjaro, Manyara, Mara, Mtwara, Njombe, Songwe, and Tabora. All the staff received a refresher training for 2-3 days before the initiation of the survey. In the 16 regions that took part in the MSMT project for the first time, all staff members from the selected HFs were trained by the project team. The training team included an experienced clinician and a laboratory expert. The training was done for 3 days and covered the study protocol, study documents such as informed consent forms (ICFs), case report forms (CRFs), and study logs. It also covered the basics of good clinical practices (GCP), good clinical laboratory practices (GCLP), and standard operating procedures (SOPs) of the project. After the training, HF staff conducted the enrolment of patients, which included providing study information, obtaining informed consent, performing clinical assessment of all eligible patients, filling the ICFs and CRFs, performing a laboratory test, and collecting samples as dried blood spots on filter papers (DBS).

The data collected included basic demographics (such as residence, age, and weight), axillary temperature, weight, history of fever and symptoms associated with malaria in the past 48 hours, history of travel in the past 30 days, and history of antimalarial medication in the past 14 days. Testing for malaria was done for all enrolled patients using RDTs and three brands were used in different regions/councils. All RDTs were Pf/Pan malaria and included Bioline™ Malaria Ag P.f/Pan (Abbott Diagnostics Korea, Inc., Gyeonggi-do, Korea), First Response (Premier Medical Corporation Pvt. Ltd., Gujarat, India), and SD Bioline (Standard Diagnostic Inc, Suwon, Korea). The testing process was done following routine procedures for outpatients per the manufacturer’s instructions. All the information, including RDT results, was recorded on CRFs. Each HF provided a daily report to the project team through a WhatsApp group that was set up for all HFs in the region. The report presented the number of patients screened and enrolled daily and the number of patients with positive results who were recruited. The report also presented the status and progress of the survey and the challenges encountered, if any. In case the staff at the HF had critical problems, such as a limited understanding of study procedures, which limited the progress of the survey, they were re-trained virtually or visited by the project team.

Quality control processes included daily and weekly reviews of the data collected at each HF by the local team itself or with the support of the MSMT team (virtually). This was done to ensure the study documents (ICF, CRF and logs) were appropriately and correctly filled, and the documents were properly stored. To further ensure high-quality data and samples were collected, each HF was supervised by the MSMT team at least twice after the training. The supervision was done before the HF team collected half of the target samples and during the closure of the survey, once all the samples were collected. During supervision, the project team assessed the progress of the survey to ensure compliance with the protocol, GCP/GCLP and SOPs. The study team assessed all study documents and samples, identifying and resolving any discrepancies. The storage of samples and data was also evaluated, along with stock levels, to ensure sufficient materials were available at the facility. In case poor samples or data were collected, they were rejected, and the team was retrained and advised to restart the sample and data collection process.

### Data management and statistical analysis

Data were collected using paper questionnaires (the CRF) and double-entered into a Microsoft ^®^ Access ^®^ LTSC MSO (Version 2403) database. Data cleaning involved exporting raw data to the Microsoft ^®^ Excel ^®^ LTSC MSO (Version 2403), removing duplicate entries, and handling missing values. Cleaned data were processed and analysed using STATA Software version 13 (StataCorp LP, College Station, TX, USA). A descriptive analysis, including median, frequencies, and proportions, was done to understand the data’s basic patterns and distributions. Inferential statistics involved chi-square and logistic regression to determine the statistical association between NMFIs and risk variables such as sex, age group, and transmission strata of malaria. All variables with a p-value < 0.25 in the univariate logistic regression were included in the multivariate regression model to understand their individual and combined effects while controlling for potential confounding factors. The association between variables were presented as crude (cORs) and adjusted odds ratios (aORs), with 95% confidence intervals (CIs), and a p-value of ≤ 0.05 was considered significant. Maps were created using *sf* package in R version 4.3.2 (The R Foundation for Statistical Computing, Vienna, Austria).

## Results

### Baseline characteristics of study patients

A total of 18,568 patients were recruited between January 2023 and August 2023, from 86 HFs across all 26 regions of Mainland Tanzania. The majority (57.8%, n = 10,724/18,568) of the patients were females while 42.2%, (n = 7,844/18,568) were males, and this pattern was observed in all regions except in Iringa region where 50.5% (n = 48/95) were male and 49.5% (n = 47/95) were female. The median age (interquartile range) of the patients was 9.0(3 – 26) years, with a high number of patients aged ≥ 15 years old (41.8%, n = 7,762/18,568), followed by under-fives (41.4%, n = 7,684/18,568), and school children (aged 5 - < 15 years) (16.8%, n = 3,122/18,568). The average number of patients per region was 714, but Dar es Salaam, Kagera, Kilimanjaro, and Tabora regions contributed over 30.0% of the study patients with ≥1,004 patients per region. All the remaining regions had over 400 patients except Iringa region which had 95 patients only (recruitment was done by one HF due to a lack of facilities with a sufficient number of patients). Based on the strata of malaria transmission intensities, 17.8% (n = 3,311/18,568) of the patients were from very low, 16.0% (n = 2,979/18,568) from low, 31.8% (n = 5,906/18,568) from moderate and 34.3% (n = 6,372/18,568) were from regions located within the high transmission strata ( Table 1).

**Table 1.** Demographic characteristics of the study patients.

Region/transmission strata	N	Male	Female	< 5 years	5 -<15 years		≥ 15 years	
		n (%)	n (%)	n (%)	n (%)		n (%)	
**Very low**								
Arusha	561	259 (46.2)	302 (53.8)	198 (35.3)	94 (16.8)		269 (47.9)	
Iringa	95	48 (50.5)	47 (49.5)	44 (46.3)	26 (27.4)		25 (26.3)	
Kilimanjaro	2154	882 (40.9)	1,272 (59.1)	308 (14.3)	238 (11.1)		1,608 (74.6)	
Manyara	596	261 (43.8)	335 (56.2)	165 (27.7)	113 (18.9)		318 (53.4)	
Njombe	402	177 (44.0)	225 (56.0)	102 (25.4)	104 (25.9)		196 (48.7)	
**Sub-Total **	**3808**	**1,627 (42.7)**	**2,181 (57.3)**	**817 (21.5)**	**575 (15.0)**		**2,416 (63.5)**	
**Low**								
Dar-es-salaam	1117	558 (50.0)	559 (50.0)	340 (30.4)	161 (14.4)		616 (55.2)	
Dodoma	841	359 (42.7)	482 (57.3)	320 (38.1)	138 (19.4)		383 (44.5)	
Singida	524	226 (43.1)	298 (56.9)	341 (65.1)	51 (9.7)		132 (25.2)	
Songwe	464	195 (42.0)	269 (58.0)	156 (33.6)	118 (25.4)		190 (40.0)	
**Sub-Total **	**2946**	**1,368 (45.9)**	**1,611 (54.1)**	**1,157(39.3)**	**468 (15.9)**		**1,321 (44.8)**	
**Moderate**								
Mara	907	382 (42.1)	525 (57.9)	246 (27.1)	270 (29.8)		391 (43.1)	
Mbeya	560	228 (40.7)	332 (59.3)	208 (37.1)	82 (14.6)		270 (48.2)	
Morogoro	476	161 (33.8)	315 (66.2)	184 (38.7)	98 (20.6)		194 (40.7)	
Mwanza	455	204 (44.8)	251 (55.2)	222 (48.8)	106 (23.3)		127 (27.9)	
Pwani	667	287 (43.0)	380 (57.0)	315 (47.2)	121 (18.1)		231 (34.6)	
Rukwa	662	265 (40.0)	397 (60.0)	267 (40.3)	150 (26.7)		245 (37.0)	
Simiyu	477	171 (35.8)	306 (64.2)	250 (52.4)	74 (15.5)		153 (32.1)	
Shinyanga	430	180 (41.9)	250 (58.1)	219 (50.9)	97 (22.6)		114 (26.5)	
Tabora	1004	435 (43.3)	569 (56.7)	605 (60.2)	1015 (10.1)		273 (29.7)	
Tanga	553	205 (37.1)	348 (62.9)	139 (25.1)	141 (25.5)		273 (49.4)	
**Sub-Total **	**6191**	**2,518 (40.7)**	**3,673 (59.3)**	**2,655 (42.9)**	**1,240 (20.0)**		**2,296 (37.1)**	
**High**								
Geita	461	200 (43.4)	261 (56.6)	245 (53.2)	95 (20.6)		121 (26.3)	
Kagera	2352	992 (42.2)	1,360 (57.8)	1,333 (56.7)	328 (13.9)		691 (29.4)	
Katavi	578	201 (34.8)	377 (65.2)	201 (34.8)	107 (18.5)		270 (46.7)	
Kigoma	460	199 (43.3)	261 (56.7)	240 (52.2)	60 (13.0)		160 (34.8)	
Lindi	426	175 (41.1)	251 (58.9)	269 (63.2)	49 (11.5)		108 (25.3)	
Mtwara	820	348 (42.1)	472 (57.6)	522 (63.7)	78 (9.5)		220 (26.8)	
Ruvuma	526	246 (46.8)	280 (53.2)	245 (46.6)	122 (23.2)		159 (30.2)	
**Sub-Total **	**5623**	**2,361 (42.0)**	**3,262 (58.0)**	**3,055 (54.3)**	**839 (14.9)**		**1,729 (30.75)**	
**Grand total**	**18568**	**7,844 (42.2)**	**10,724 (57.8)**	**7,684 (41.4)**	**3,122 (16.8)**		**7,762 (41.8)**	

### Prevalence of non-malarial febrile illnesses

**Figure 3.**
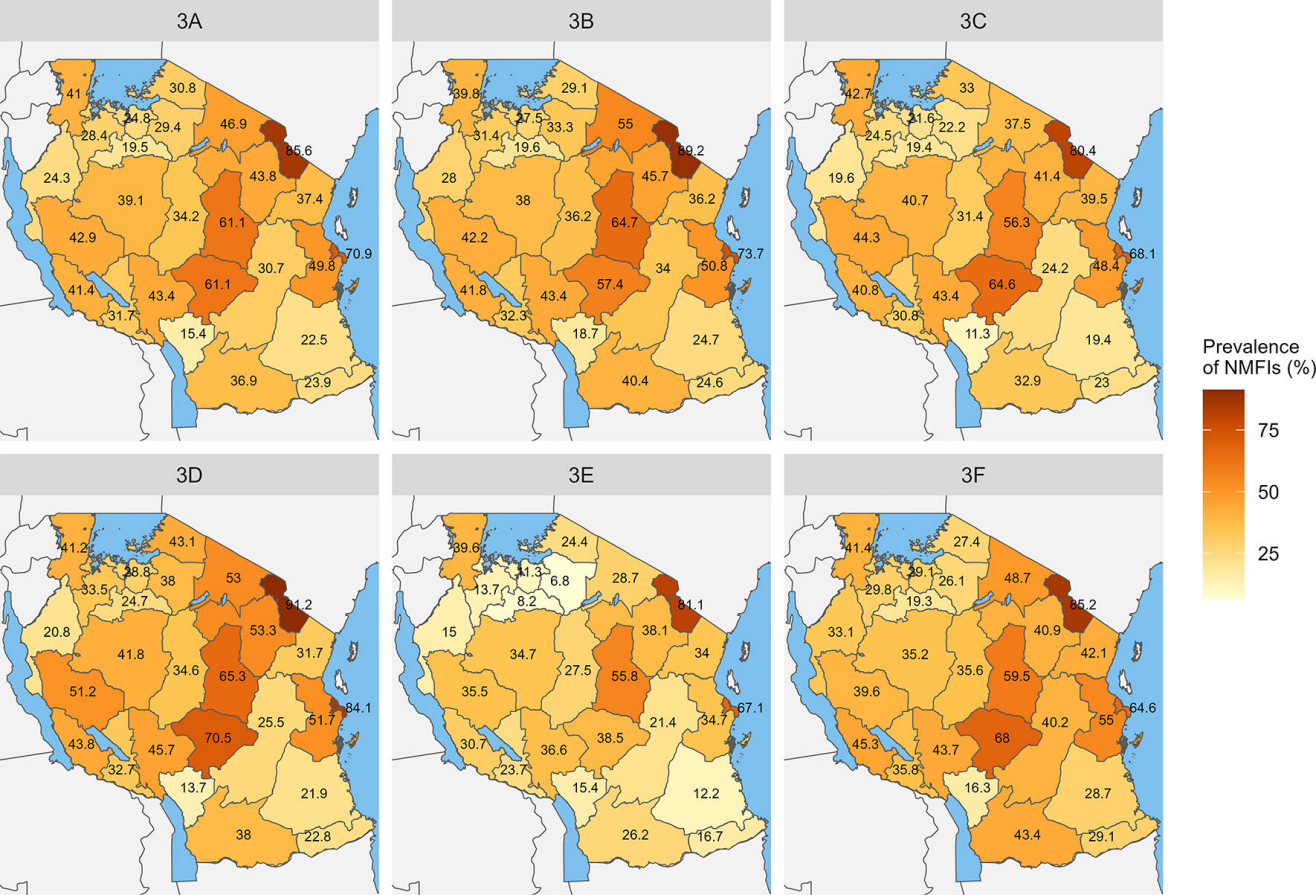
Spatial distribution of non-malarial febrile illness prevalence in six demographic groups: (3A) all patients, (3B) females, (3C) males, (3D) under-fives, (3E) school children, and (3F) adults. Each panel highlights the variation in NMFI prevalence across the study region for the specified subgroup.

Of the 18,568 patients tested, 8,273 (44.6%) had a negative test for malaria by RDT and were considered to have NMFIs ( Table 2). Of all regions, Dar-es-salaam and Kilimanjaro had the highest prevalence of NMFIs (70.9%, n = 792/1,117 and 85.6%, n = 1,844/2,154, respectively) while Njombe had the lowest prevalence with 15.4% (n = 62/402) ( Figure 3A). Regional variation in sample size may have influenced the precision of these estimates. The prevalence of NMFIs was significantly higher in females (45.8%, n = 4,913/10,724) than in males (42.8%, n = 3,360/7,844). This pattern was observed in all regions except in Iringa, where the prevalence was higher in males (64.6%, n = 31/48) than in females (57.5%, n = 27/47). The proportion of NMFI was significantly higher in adults (50.6%, n = 3,927/7,762) compared to under-fives (42.6%, n = 3,276/7,684) and school children (34.3%, n = 1,070/3,122, respectively). In the male population, the high prevalence was observed in Kilimanjaro (80.4%, n = 709/882), Dar-es-Salaam (68.1%, n = 380/558), and Iringa (64.6%, n = 31/48), while Kigoma, Shinyanga, Lindi and Njombe regions had lower prevalence with 19.6% (n = 39/199), 19.4% (n = 35/180), 19.4% (n = 34/173) and 11.3% (n = 20/177), respectively. Among females, Kilimanjaro (89.2%, n = 1,135/1,272), Dar es Salaam (73.7%, n = 412/559), and Dodoma (64.7%, n = 312/482) also had a high prevalence of NMFIs, reflecting the trend observed in males. Regions such as Lindi (24.7%, n = 62/251), Mtwara (24.6%, n = 116/472), Shinyanga (19.6%, n = 49/250), and Njombe (18.7%, n = 42/225) had lower prevalence in the female population ( Figure 3B & 3C).

**Table 2.** Prevalence of NMFI by sex, age group, and malaria transmission strata.

Variable	Febrile patients tested for malaria	Patients with negative malaria tests	Proportion of NMFI (%)
Overall prevalence	18,568	8,273	44.6
Sex			
Male	7,844	3,360	42.8
Female	10,724	4,913	45.8
Age group			
<5 years	7,684	3,276	42.6
5 -< 15 years	3,122	1,070	34.3
≥15 years	7,762	3,927	50.6
Transmission strata			
Very Low	3,808	2,488	65.3
Low	2,946	1,632	55.4
Moderate	6,191	2,211	35.7
High	5,623	1,942	34.5

**Figure 4.**
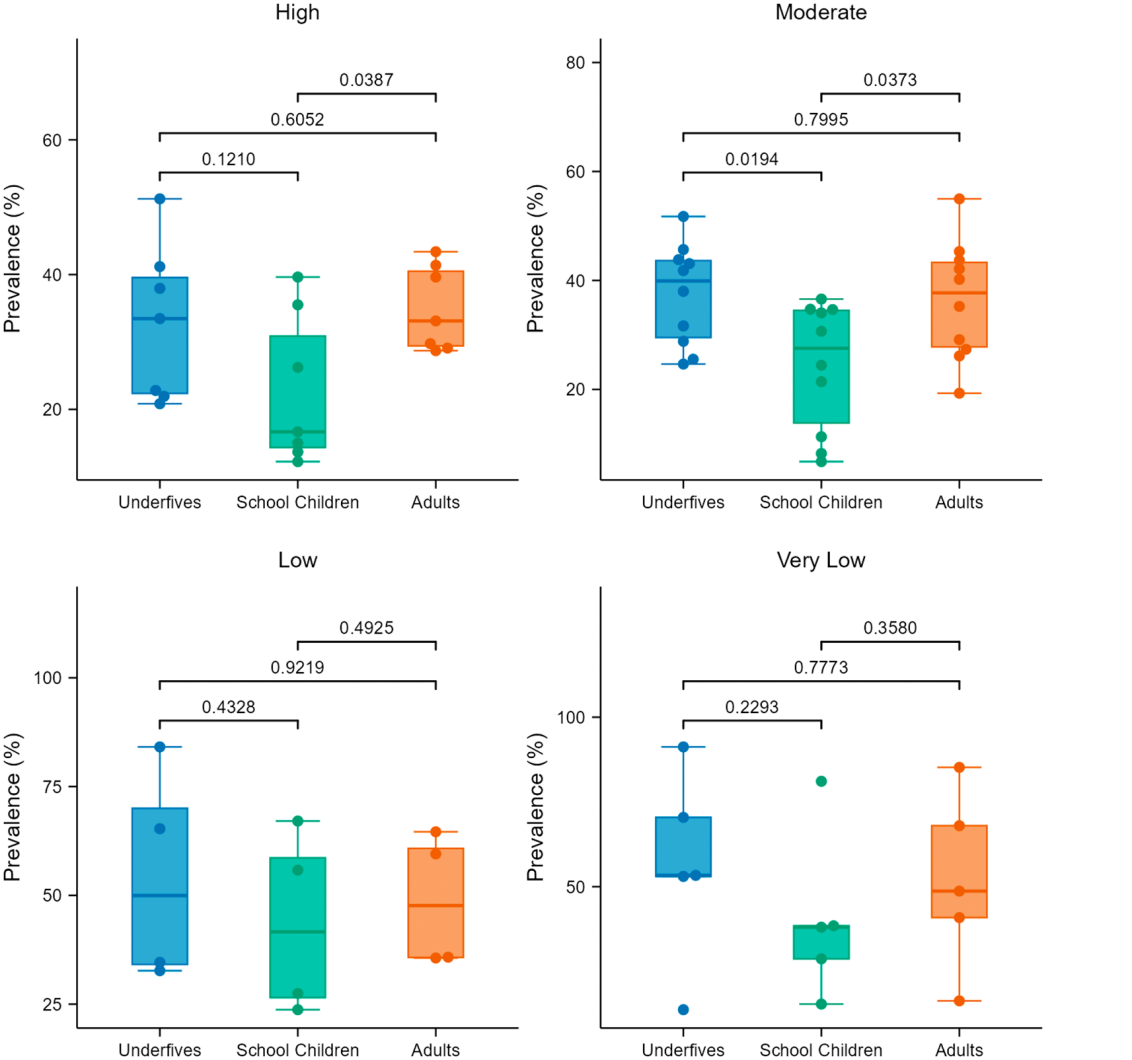
Boxplots showing the prevalence of NMFIs among patients of different age groups in the strata with different malaria transmission intensities.

In under-fives, the regions of Kilimanjaro (91.2%, n = 281/308), Dar-es-Salaam (84.1%, n = 286/340), Iringa (70.5%, n = 31/44) and Dodoma (65.3%, n = 209/320) had a higher prevalence of NMFIs, indicating a significant burden of NMFIs among young children. On the contrary, regions like Njombe (13.7%, n = 14/102), Kigoma (20.8%, n = 50/240), and Lindi (21.9%, n = 59/269) had a lower prevalence of NMFIs in under-fives. Among school children, Kilimanjaro (81.1%, n =193/238) and Dar-es-Salaam (67.1%, n = 108/161) had a higher prevalence of NMFIs, while Simiyu (6.8%, n = 5/74), Shinyanga (11.3%, n = 8/97), and Mwanza (11.3%, n = 12/106) had lower prevalence in this age group. In the adult population, the highest prevalence was observed in Kilimanjaro region (85.2%, n = 1,370/1,608), while Njombe (16.3%, n = 32/196) had the lowest prevalence ( Figure 3D-F and Supplementary Table 1). The proportion of NMFI was relative lower in the high transmission stratum (34.5%) compared to the very low transmission stratum (65.3%), as shown in Figure 4.

### Predictors/risk factors of non-malarial illnesses

In the univariate analysis, sex, age group, and malaria transmission strata were significantly associated with the occurrence of NMFIs (p < 0.001). When all these factors were simultaneously included in a multivariate model, the odds of getting NMFI were significantly higher in females compared to males (adjusted odds ratio [aOR] = 1.19, 95% CI = 1.10–1.26, p < 0.001). Compared to adults, the odds of getting NMFI were significantly higher in under-fives (aOR = 1.17, 95% CI = 1.09–1.26, p < 0.001), while school children had less likelihood of infections (aOR = 0.70, 95% CI = 0.63–0.77, p < 0.001). Moreover, the odds of having NMFIs were significantly lower in individuals living in settings with moderate (aOR = 0.09, 95% CI = 0.08 – 0.12, p < 0.001), low (aOR = 0.07, 95% CI = 0.06–0.09, p < 0.001) and high transmission intensities of malaria (aOR = 0.11, 95% CI = 0.08-0.13, p < 0.001); compared to those living in the settings with very low transmission intensities ( Table 3).

**Table 3.** Demographic factors associated with NMFIs.

Variable	Crude OR	95% CI	P value	Adjusted OR	95% CI	P value
Sex						
Female	1.13	1.06–1.20	<0.001	1.18	1.10–1.26	<0.001
Male	Reference			Reference		
Age group						
< 5 years	1.43	1.31–1.55	<0.001	1.17	1.09–1.26	<0.001
5 –<15 years	0.51	0.47–0.56	<0.001	0.70	0.64–0.77	<0.001
≤ 15 years	Reference			Reference		
Transmission strata						
Very Low	Reference			Reference		
Low	0.66	0.59–0.73	<0.001	0.07	0.06–0.09	<0.001
Moderate	0.29	0.27–0.32	<0.001	0.09	0.08–0.12	<0.001
High	0.28	0.26–0.31	<0.001	0.11	0.08–0.13	<0.001
Type of RDT						
Abbott Bioline	1.44	1.35–1.53	<0.001	1.33	1.21–1.45	<0.001
SD Bioline	1.84	1.63–2.09	<0.001	1.92	1.63–2.27	<0.001
First Response	Reference			Reference		

## Discussion

This was the first health facility-based cross-sectional study involving symptomatic patients suspected of having uncomplicated malaria, conducted by the MSMT project, covering all 26 regions of Mainland Tanzania. Unlike community surveys, which are routinely implemented in school children, ^39^ or under-fives. ^11^^,^^40^^–^^42^ It provides the largest scan of malaria in HF settings across the entire country. The data have also been utilised to assess the prevalence and patterns of NMFIs in Tanzania and provide useful information for guiding policy and decisions for controlling different pathogens associated with NMFIs. The study found that more than 40.0% of febrile patients had NMFIs, and there was a high prevalence of NMFIs with spatial heterogeneity across different regions, characterized by varying malaria transmission intensities in Mainland Tanzania. Occurrence and the odds of NMFIs were higher in females than in males, and in adults compared to younger age groups. School children had the lowest prevalence and, correspondingly, the lowest odds of getting NMFIs. NMFIs varied inversely to malaria transmission intensities, with the lowest prevalence in the regions located in the stratum with high transmission intensities and the highest prevalence in the stratum with very low transmission intensities of malaria. The findings provide country-wide scans of NMFIs with varying prevalence, which suggest that more studies are urgently needed to characterise the burden of NMFIs further and develop POC tests and guidelines/recommendations for managing these illnesses alongside malaria.

The findings showed that the prevalence of NMFIs in the different regions of Mainland Tanzania increased with decreasing malaria transmission intensities. This may or may not represent a direct association between the occurrence of NMFIs and the transmission intensity of malaria. In areas with high malaria transmission, the rate of individuals harbouring malaria parasites is high compared to areas with low transmission. However, most of the people in high transmission tend to have high acquired immunity due to high rates of exposure to infective bites by mosquitoes. ^46^ Thus, in high-transmission areas, not all malaria infections cause symptoms, as some individuals may remain asymptomatic even when they have high parasitaemia. ^47^ When such individuals develop symptoms, they will test positive for malaria, while malaria may potentially not be the direct cause of the symptoms. ^13^ As a result, any individual who tests positive for malaria by RDT will be considered not to have NMFIs (based on the criteria used in this study), resulting in a low prevalence of NMFIs and potentially improper case management of infections with fever as the main symptom. On the contrary, in areas with low malaria burden, most of the febrile patients will test negative for malaria, thus reducing the masking effect of malaria infections and resulting in high NMFI prevalence. Therefore, while it may be true that in some areas where the prevalence of NMFIs is high and the burden of malaria is low, co-infections with malaria, which is common in areas of high transmission intensities, may introduce bias and act as a confounder. Thus, other causes of fever should always be investigated even in malaria-positive individuals, particularly in high malaria-burden regions, although this may be challenging under current clinical conditions with limited POC tests for other pathogens. ^13^

This study showed high variations in NMFI prevalence among patients in different age groups, with adults exhibiting a higher prevalence than under-fives and school children. The increased prevalence in adults suggests that the adult population may be more exposed to febrile illnesses due to increased mobility, occupational exposure, and sometimes immunosenescence. Taking more responsibility, adults may be exposed to multiple and higher doses of infectious agents than younger children. Increased exposure frequently raises the chance of contracting an infection, but there is no apparent evidence linking this risk to the findings of this study. ^48^ The observed higher prevalence in under-fives could be due to the vulnerability of this group to infections. These findings are similar to those reported in Malawi, where 72.0% of under-fives and 77.0% of adults had NMFIs. ^29^ The low prevalence of NMFIs among school children could be attributed to the high prevalence of malaria in this group, as previously reported by other studies, ^44^^,^^49^^,^^50^ since febrile patients were excluded from NMFI analysis if they tested positive for malaria by RDT. Additionally, the low prevalence of NMFI among school children could be attributed to their relatively stronger immunity acquired through repeated antigenic exposure. It may also be because, at the age of 5-15, the immune system is just starting to mature. As a result, most infections can be prevented by the immune system, making this group less susceptible to most NMFI-causing pathogens. ^51^^,^^52^ This is consistent with previous research conducted elsewhere, where school children had the lowest prevalence of NMFI compared to under-fives and adults. ^29^^,^^53^ The pattern observed in this study is similar to what was reported by other studies on malaria-related fevers, which showed a higher prevalence of malaria in school children, making them have NMFIs in the absence of tests for other pathogens. ^44^^,^^49^ This results in a low prevalence of non-malarial fevers since a large proportion of the fevers are parasitologically attributed to malaria, even if there is no evidence of clinical association unless multiple tests are done to rule out any other infections by fever-causing pathogens.

The slightly higher NMFI prevalence observed in females compared to males may be attributed to various social, biological, and healthcare-seeking behaviors. In many settings, women are more likely to seek healthcare services, leading to higher detection rates of febrile illnesses. Additionally, biological differences such as immune response variations between sexes may also contribute to this observation. ^54^^,^^55^ Due to social and biological factors, the prevalence of malaria is usually higher in males compared to females, ^44^^,^^49^^,^^50^ which results in the exclusion of more males than females in the NMFI analysis, since the potential for malaria co-infection with other febrile aetiologies was not pursued in this study. However, further research is needed to fully understand the underlying causes of this disparity. The findings of higher odds of NMFIs are consistent with what was reported by other studies, which showed a higher prevalence of NMFIs in females (70.0%) compared to males (68.0%). ^29^

The key strength of the study is its extensive coverage, utilising a large number of samples from all 26 regions of Mainland Tanzania, thus providing a comprehensive representation of the country’s diverse population and transmission settings. However, this study had some limitations. As a cross-sectional study, the data may not accurately represent the actual prevalence of NMFIs in a given area, as the study only captured a single time point and, therefore, had a limited temporal scope. Also, the data for this study were collected as part of the MSMT project, whose sites were purposively selected to maximise the recruitment of patients with malaria based on the detection of RDT-positive results. Therefore, the sites may be biased towards high transmission areas, even in low transmission regions, given the current heterogeneous nature of malaria in Mainland Tanzania. ^56^^,^^57^ This selection criterion, while operationally necessary to meet recruitment targets, may have underestimated the proportion of NMFIs, particularly in low-transmission settings. Thus, future studies should consider inclusive sampling covering areas of different levels of malaria burden, preferably using DHIS2 data for a more comprehensive mapping of NMFIs country-wide. Additionally, this study could not explore the specific causes of NMFIs because it was not designed to offer POC or laboratory tests for other pathogens. However, it has highlighted the magnitude of this problem in the entire country, with a higher burden of NMFIs, particularly in areas of low transmission intensities, as previously reported by other studies. ^13^ Analysis to address this issue will need to be performed and reported in future studies to appropriately inform policy and decisions for targeting and better case management, and the control of NMFIs.

## Conclusion

Non-malarial febrile illnesses were fairly common, and their burden and distribution patterns varied significantly across regions and malaria transmission strata. Females, adults, and individuals from areas with low and very low transmission intensities of malaria had a higher prevalence of NMFIs and an increased likelihood of experiencing non-malarial fevers compared to males, under-fives, school children, and individuals living in settings with high malaria transmission intensities. These findings have important implications for healthcare planning, offering critical insights to enhance the diagnosis and management of febrile illnesses beyond malaria. The results further suggest that the identified groups of patients should be appropriately targeted with POC tests and treatment strategies. By country-wide mapping of NMFIs, this research contributes to a better understanding of the local epidemiology of NMFIs and supports the development of more effective management and control strategies. Addressing the gaps in evidence-based information on NMFIs will ultimately lead to improved patient outcomes and better healthcare delivery in regions with varying endemicity of malaria and other fever-causing pathogens.

## Ethics approval and consent to participate

This large health facility survey was part of the MSMT project, whose protocol was reviewed and approved by the Medical Research Coordinating Committee (MRCC) of the National Institute for Medical Research (NIMR), on 15 November 2022, with certificate number NIMR/HR/R.8c/Vol. I/2193. Permission to conduct the study in the different regions was sought from the President’s Office, Regional Administration and Local Government (PO-RALG). Before participating in the survey, all participants were asked and provided individual consent (or assent for children aged 7–17 years of age) for their participation in the survey. For children under the legal age of adulthood in Tanzania (<18 years), consent was obtained from a parent or guardian. An informed consent form was developed in English and translated into Kiswahili and used to obtain consent both verbally and in writing from all participants. All participants agreed and signed the consent or assent form or provided a thumbprint in conjunction with the signature of an independent witness in case the study participant was illiterate.

## Disclosure

The previous version of this manuscript was published as a preprint ^58^ and original pre-preprint underwent rapid review.

## Supplementary material

**Supplementary Table 1.** Prevalence of non-malaria febrile illnesses (NMFIs) by region, sex, and age group.

Regions				Sex						Age group								
				Female			Male			<5 years			5 -< 15 years			≤15 years		
	Tested for malaria	Negative malaria tests	Proportion of NMFI (%)	Tested for malaria	Negative malaria tests	Proportion of NMFI (%)	Tested for malaria	Negative malaria tests	Proportion of NMFI (%)	Tested for malaria	Negative malaria tests	Proportion of NMFI (%)	Tested for malaria	Negative malaria tests	Proportion of NMFI (%)	Tested for malaria	Negative malaria tests	Proportion of NMFI (%)
Arusha	561	263	46.9	302	166	55.0	259	97	37.5	198	105	53.0	94	27	28.7	269	131	48.7
Dar es Salaam	1117	792	70.9	559	412	73.7	558	380	68.1	340	286	84.1	161	108	67.1	616	398	64.6
Dodoma	841	514	61.1	482	312	64.7	359	202	56.3	320	209	65.3	138	77	55.8	383	228	59.5
Geita	461	131	28.4	261	82	31.4	200	49	24.5	245	82	33.5	95	13	13.7	121	36	29.8
Iringa	95	58	61.1	47	27	57.4	48	31	64.6	44	31	70.5	26	10	38.5	25	17	68.0
Kagera	2352	965	41.0	1360	541	39.8	992	424	42.7	1333	549	41.2	328	130	39.6	691	286	41.4
Katavi	578	248	42.9	377	159	42.2	201	89	44.3	201	103	51.2	107	38	35.5	270	107	39.6
Kigoma	460	112	24.3	261	73	28.0	199	39	19.6	240	50	20.8	60	9	15.0	160	53	33.1
Kilimanjaro	2154	1844	85.6	1272	1135	89.2	882	709	80.4	308	281	91.2	238	193	81.1	1608	1370	85.2
Lindi	426	96	22.5	251	62	24.7	175	34	19.4	269	59	21.9	49	6	12.2	108	31	28.7
Manyara	596	261	43.8	335	153	45.7	261	108	41.4	165	88	53.3	113	43	38.1	318	130	40.9
Mara	907	279	30.8	525	153	29.1	382	126	33.0	246	106	43.1	270	66	24.4	391	107	27.4
Mbeya	560	243	43.4	332	144	43.4	228	99	43.4	208	95	45.7	82	30	36.6	270	118	43.7
Morogoro	476	146	30.7	315	107	34.0	161	39	24.2	184	47	25.5	98	21	21.4	194	78	40.2
Mtwara	820	196	23.9	472	116	24.6	348	80	23.0	522	119	22.8	78	13	16.7	220	64	29.1
Mwanza	455	113	24.8	251	69	27.5	204	44	21.6	222	64	28.8	106	12	11.3	127	37	29.1
Njombe	402	62	15.4	225	42	18.7	177	20	11.3	102	14	13.7	104	16	15.4	196	32	16.3
Pwani	667	332	49.8	380	193	50.8	287	139	48.4	315	163	51.7	121	42	34.7	231	127	55.0
Rukwa	662	274	41.4	397	166	41.8	265	108	40.8	267	117	43.8	150	46	30.7	245	111	45.3
Ruvuma	526	194	36.9	280	113	40.4	246	81	32.9	245	93	38.0	122	32	26.2	159	69	43.4
Shinyanga	430	84	19.5	250	49	19.6	180	35	19.4	219	54	24.7	97	8	8.2	114	22	19.3
Simiyu	477	140	29.4	306	102	33.3	171	38	22.2	250	95	38.0	74	5	6.8	153	40	26.1
Singida	524	179	34.2	298	108	36.2	226	71	31.4	341	118	34.6	51	14	27.5	132	47	35.6
Songwe	464	147	31.7	269	87	32.3	195	60	30.8	156	51	32.7	118	28	23.7	190	68	35.8
Tabora	1004	393	39.1	569	216	38.0	435	177	40.7	605	253	41.8	101	35	34.7	298	105	35.2
Tanga	553	207	37.4	348	126	36.2	205	81	39.5	139	44	31.7	141	48	34.0	273	115	42.1
**Total**	**18568**	**8273**	**44.6**	**10724**	**4913**	**45.8**	**7844**	**3360**	**42.8**	**7684**	**3276**	**42.6**	**3122**	**1070**	**34.3**	**7762**	**3927**	**50.6**

## Data Availability

The data supporting this study are available upon reasonable request from the corresponding author (MDS), subject to institutional approval by the Medical Research Coordinating Committee (MRCC) of the National Institute for Medical Research (NIMR), and a signed data transfer agreement. Data cannot be publicly shared due to ethical considerations. Access will be granted in accordance with the MRCC approval.
